# Methylation Warfare: Interaction of Pneumococcal Bacteriophages with Their Host

**DOI:** 10.1128/JB.00370-19

**Published:** 2019-09-06

**Authors:** Leonardo Furi, Liam A. Crawford, Guillermo Rangel-Pineros, Ana S. Manso, Megan De Ste Croix, Richard D. Haigh, Min J. Kwun, Kristine Engelsen Fjelland, Gregor D. Gilfillan, Stephen D. Bentley, Nicholas J. Croucher, Martha R. Clokie, Marco R. Oggioni

**Affiliations:** a Department of Genetics and Genome Biology, University of Leicester, Leicester, United Kingdom; b MRC Centre for Global Infectious Disease Analysis, Department of Infectious Disease Epidemiology, Imperial College London, London, United Kingdom; c Department of Medical Genetics, Oslo University Hospital and University of Oslo, Oslo, Norway; d Parasites and Microbes, Wellcome Trust Sanger Institute, Hinxton, United Kingdom; Rutgers University-Robert Wood Johnson Medical School

**Keywords:** DNA methylation, *Streptococcus pneumoniae*, abortive infection, bacteriophage genetics, phase variation, restriction-modification system

## Abstract

Virus-host interactions are regulated by complex coevolutionary dynamics. In Streptococcus pneumoniae, phase-variable type I restriction-modification (R-M) systems are part of the core genome. We hypothesized that the ability of the R-M systems to switch between six target DNA specificities also has a key role in preventing the spread of bacteriophages. Using the streptococcal temperate bacteriophage SpSL1, we show that the variants of both the SpnIII and SpnIV R-M systems are able to restrict invading bacteriophage with an efficiency approximately proportional to the number of target sites in the bacteriophage genome. In addition to restriction of lytic replication, SpnIII also led to abortive infection in the majority of host cells. During lytic infection, transcriptional analysis found evidence of phage-host interaction through the strong upregulation of the *nrdR* nucleotide biosynthesis regulon. During lysogeny, the phage had less of an effect on host gene regulation. This research demonstrates a novel combined bacteriophage restriction and abortive infection mechanism, highlighting the importance that the phase-variable type I R-M systems have in the multifunctional defense against bacteriophage infection in the respiratory pathogen S. pneumoniae.

**IMPORTANCE** With antimicrobial drug resistance becoming an increasing burden on human health, much attention has been focused on the potential use of bacteriophages and their enzymes as therapeutics. However, the investigations into the physiology of the complex interactions of bacteriophages with their hosts have attracted far less attention, in comparison. This work describes the molecular characterization of the infectious cycle of a bacteriophage in the important human pathogen Streptococcus pneumoniae and explores the intricate relationship between phase-variable host defense mechanisms and the virus. This is the first report showing how a phase-variable type I restriction-modification system is involved in bacteriophage restriction while it also provides an additional level of infection control through abortive infection.

## Abstract (precis)

With antimicrobial drug resistance becoming an increasing burden on human health, much attention has been focused on the potential use of bacteriophages and their enzymes as therapeutics. However, the investigations into the physiology of the complex interactions of bacteriophages with their hosts have attracted far less attention, in comparison. This work describes the molecular characterization of the infectious cycle of a bacteriophage in the important human pathogen Streptococcus pneumoniae and explores the intricate relationship between phase-variable host defense mechanisms and the virus. This is the first report showing how a phase-variable type I restriction-modification system is involved in bacteriophage restriction while it also provides an additional level of infection control through abortive infection.

## INTRODUCTION

In almost all ecosystems studied, bacteria are vastly outnumbered by coexisting bacteriophages (1). The survival of both is sustained by an endless state of coevolutionary cycles of adaptation and counteradaptation (2). Bacteria have therefore evolved many resistance mechanisms to limit phage infection, including the inhibition of phage attachment to the cell, cleavage of the invading phage genome, and altruistic programmed cell death to abort phage infection. In response, phages evolved the so-called host range-expanding adaptations. This continuous selection for defense and counterdefense traits is often described as an “arms race” (3).

Streptococcus pneumoniae, the pneumococcus, is one of the most important human pathogens, being the major cause of community-acquired pneumonia, meningitis, and acute otitis media (4, 5). Interestingly, most sequenced S. pneumoniae strains contain prophage genes which sum to approximately 6% of the pneumococcal genome (6), with 30% of all pneumococcal strains harboring an intact prophage within their genomes (6). Despite this apparent pneumococcal phage abundance, only a few bacteriophages have been characterized and studied, including DP-1, CP-1, and the temperate phage MM1 (7–10). It is only recently that pneumococcal phage biology has again attracted interest, with the majority of research focusing on the use of their endolysins as potential therapeutics (7–10), as opposed to their genomics and their influence on the biology of the pneumococcus (11–13). However, recent genome-wide association studies (GWAS) have indicated that phages have a strong effect on their host cells’ epidemiology. One found that prophages disrupting the *comYC* genes were asymptomatically carried for durations shorter than expected (14), and the second showed a strong correlation between 30-day patient mortality from S. pneumoniae-related sepsis and the gene for the phage tail fiber protein (PblB), a protein shown to bind and activate platelets (15). Even though pneumococcal prophages show a high degree of genetic heterogeneity (6, 16), this PblB tail fiber protein is present in 72% of all sequenced pneumococcal bacteriophages (6).

**FIG 1:**
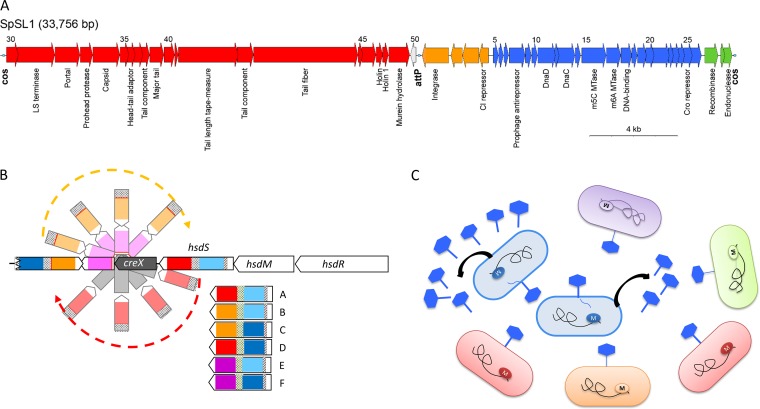
The SpSL1 bacteriophage and *spnIII* restriction system. (A) The SpSL1 genome displayed in the viral conformation with the cohesive ends flanking the sequence. The color scheme indicates the operon structure, with operon 1 (*cds1* to *cds4*) in orange, operon 2 (*cds5* to *cds26*) in blue, operon 3 (*cds27* to *cds29*) in green, operon 4 (*cds30* to *cds49*) in red, and operon 5 (*cds50*) in white (GenBank accession number KM882824). (B) The phase-variable type I restriction-modification system *spnIII* containing the *hsdR*, *hsdM*, and variable *hsdS* genes, in addition to the site-specific recombinase gene *creX*, with a schematic representation of the recombination. The *hsdS* gene encodes N-terminal and C-terminal target recognition domains (TRDs). The two N-terminal TRDs are in dark blue and light blue, while the C-terminal TRDs are in red, orange, and purple. The inverted repeats are shown as gray dotted rectangles. The six different *hsdS* variants, indicated A to F, are shown below (20). (C) A cartoon for a population-based bacteriophage defense that arises from a phase-variable restriction-modification system, where the bacterial genome is methylated in a specific pattern (shown by the colored M on the black line). The bacteriophage would be able to infect and replicate in only one variant (blue), while it would be unable to infect the other variants with different methylation patterns (orange, red, purple, and green) (21).

Phage defense mechanisms in S. pneumoniae primarily rely on a panel of restriction-modification (R-M) systems (17). Strains harbor, alternatively, one of the three allelic variants of the DpnI, DpnII, or DpnIII type II R-M systems (18), one or two further type II R-M systems, and two conserved phase-variable type I R-M systems (19, 20). Among them, the type I R-M system SpnIII is most conserved, existing in almost all S. pneumoniae isolates (19, 21). The *spnIII* operon comprises a contingency locus (22), defined as the inverting variable restriction (*ivr*) locus (19), in which high-frequency rearrangements occur between the active *hsdS* and two other untranscribed *hsdS*-like genes (19, 20, 23). These recombination events lead to the alternate formation of six *hsdS* genes with different DNA sequence specificities that coexist in a bacterial population (Fig. 1B) (20). The variability of SpnIII has been hypothesized to have a role in preventing phage transmission in clonally related bacterial populations (Fig. 1C) (19, 21). The presence of the different enzyme forms should prevent the spread of phages within populations, even within the same strain. Once a bacteriophage has infected bacteria with one variant of the SpnIII system, its susceptibility to restriction from the other five variants would theoretically be unchanged in comparison with that of a naive phage (Fig. 1C). Despite this, experimental demonstrations of the functionality of this R-M system in double-stranded DNA cleavage and in phage resistance are still lacking.

Another one of the well-studied bacteriophage defense mechanism is the induction of altruistic programmed cell death of a phage-infected cell; this process has been defined as an abortive infection (Abi) or a phage exclusion system. These systems promote the death of infected cells in order to abort phage replication and limit its further spread within the population. Currently, only a few examples of toxin-antitoxin (TA) systems that protect bacteria from phages have been described (24–28) and are considered a subgroup of the Abi systems (29). Recently, R-M systems have been compared to TA systems (30), as they can also trigger postsegregational killing (31–33) and have been shown to be important in bacteriophage defense. The type IV McrBC R-M system, previously characterized in Escherichia coli, specifically recognizes the R^m^C (R = A or G; ^m^C = ^m4^C, ^m5^C, or ^hm5^C) pattern and cleaves the DNA between two recognition sites *in vitro*. Interestingly, McrBC is known to induce Abi upon bacteriophage infection (34, 35). The McrBC system has also been shown to act as a bacteriophage defense in S. mitis, reducing the rate of DNA replication of the lytic phage DP-1 (36).

Here we report the isolation and characterization of a streptococcal temperate bacteriophage, SpSL1; this was then used as a tool to confirm the methylation and restriction activities of the phase-variable SpnIII system in S. pneumoniae. Phage-host interactions were also evaluated by means of RNA sequencing analysis of bacterial and phage genome transcriptomes. Finally, we show that the SpnIII system is involved in programmed cell death via an Abi mechanism that inhibits the proliferation of the SpSL1 bacteriophage with a different SpnIII methylation pattern.

## RESULTS

### Isolation of a temperate pneumococcal bacteriophage.

We have previously characterized a phase-variable pneumococcal type I restriction system (20). To investigate its impact on bacteriophage control, we used an *spnIII* deletion mutant as a potential permissive bacteriophage host. Screening of 24 oral swab samples from healthy adult volunteers, using encapsulated and unencapsulated mutants carrying a deleted *spnIII* locus (*spnIII*-deleted mutants) as hosts, gave a single positive spot assay result after 3 days of enrichment for the unencapsulated strain only. The phage, named SpSL1, was able to form clear plaques in a double-layer agar (d-LA) assay (see Fig. S1A in the supplemental material; all supplemental material described in this article may be found at https://doi.org/10.25392/leicester.data.8320871). A single clone was isolated and propagated to 4 × 10^9^ PFU/ml. To further characterize SpSL1, the phage morphology was determined using transmission electron microscopy (TEM) (Fig. S1B). The presence of an isometric capsid (∼50 nm) and long noncontractile tail (∼160 nm) ending in a single tail fiber (∼110 nm) revealed that SpSL1 belongs to the *Siphoviridae* family. Phage adsorption to S. pneumoniae was found to be extremely rapid. Indeed, the number of free phages in solution decreased by 98% in less than 60 seconds when they were in contact with pneumococcal cells (Fig. S1C). The adsorption rate constant during the first minute of incubation was 3.9 × 10^−8^ ml/min.

### Phage SpSL1 sequence and proteome.

Sequencing of SpSL1 (GenBank accession number KM882824) revealed a linear genome of 33,756 bp with a GC content of 38.6%. An 11-base single-stranded cohesive end (5′-CGGTGTCAATC-3′), required for genome recircularization, was found at the genome ends. The 50 predicted coding sequences (CDSs) are organized in five operons with packaging, morphogenesis, lysis, lysogeny, and replication functions (Fig. 1A). All genes are transcribed from the same strand, with the exception of those belonging to the lysogeny cluster and *cds50*, encoding an unknown hypothetical protein (Fig. 1A). Packaging and morphology modules are well conserved with respect to other pneumophages, including the tail fiber gene (*cds44*), encoding a 1,602-amino-acid (aa) PblB-like protein. The PblB-like protein is predicted to be the tail fiber of SpSL1 and shows similarity (60 to 80% amino acid identity) to bacterial platelet-binding proteins and other streptococcal bacteriophage PblB-like proteins. As in other streptococcal phages previously described in a worldwide panel of 482 pneumococcal genomes (6), the lysis gene cluster, carrying two holins and an endolysin, was present downstream of the packaging genes (Fig. 1A). The lysogeny module, located downstream of the *attP* site, comprises a 382-aa integrase belonging to the Int family (tyrosine recombinases) with an amino acid identity of about 95% compared to that in other sequenced streptococcal temperate bacteriophages (Fig. 1A). SpSL1 shows a high degree of similarity to the B2 cluster of pneumophages previously characterized (6). A transcription regulator (*cI*) and a prophage antirepressor protein are also present on this module (Fig. 1A). Of particular interest is the position of a second transcriptional regulator, *cro* (*cds25*), which is inside the replication gene cluster (Fig. 1A). The genes in this module mainly encode proteins involved in the regulation of phage replication within the host cell. In comparison to the replication modules found in other referenced pneumophages (6), the replication module in SpSL1 showed a large degree of rearrangements. In addition, the replication gene cluster also encodes two methyltransferases (MTases). Indeed, through sequence analysis, *cds15* and *cds16* are predicted to encode C-5 cytosine and N-6 adenine MTases. The methylation pattern of the m5C MTase was identified to be R^5m^ CGRC (the underlined C indicates the methylated cysteine), but we could not identify any adenine methylation. Bisulfite methylome analysis showed that 102 out of 113 RCGRC sites were methylated in the SpSL1 genome. The remaining nine nonmethylated sites are reported in Table 1, and intriguingly, these included a site in an inverted repeat in overlapping a possible promoter for the m5C MTase gene itself.

**TABLE 1:** Nonmethylated cytosines in the R^m5^CRGC pattern of the SpSL1 genome

Position	Strand	Gene	Function
214		*cds30*	Hypothetical protein
3605	c^*a*^	*cds33*	Prohead maturation protease
3661	c	*cds33*	Prohead maturation protease
18433		*cds49*	LytA-like murein hydrolase
18804	c	*cds49*	Hypothetical protein
19917	c	*cds01*	Integrase
19947	c	*cds01*	Integrase
24773	c	*cds11*	DNA replication initiation protein (DnaD)
26775	c	Intergenic	Hairpin in front of the m5C MTase (*cds15*)

a c, complementary strand.

Proteomic analysis by liquid chromatography coupled with tandem mass spectrometry (LC-MS/MS) of the SpSL1 particles revealed the presence of 8 out of the 17 predicted phage structural proteins (Table S2). Except for the hypothetical protein Cds21, none of the other nonstructural proteins yielded significant counts in the mass spectrometry (MS) analysis (Table S2).

### Phage integration attachment site.

The phage attachment sequence was found to be 5′-CTTTTTCATAATAATCTCCCT-3′. This sequence in the S. pneumoniae reference D39 genome maps to the conserved stretch of four noncoding small RNAs called *cia*-dependent small RNA 3 (csRNA3), csRNA2, csRNA4, and csRNA5 (Fig. S2A). Lysogenic SpSL1 in the *spnDP1004III*-deleted mutant was identified to be integrated in both csRNA3 and -2 (Fig. S2C and D). Despite this, the lysogens were not stable, as confirmed by PCR amplification of the phage genome in its lytic form and intact csRNAs (Fig. S2C and D). When assaying single-colony isolates of the lysogenic clones for integrated phage, SpSL1 was always found to be integrated into csRNA3, and in half of the colonies, it was also integrated into csRNA2 (data not shown). There was no evidence for specialized transduction.

### Gene expression profiling of SpSL1.

**FIG 2:**
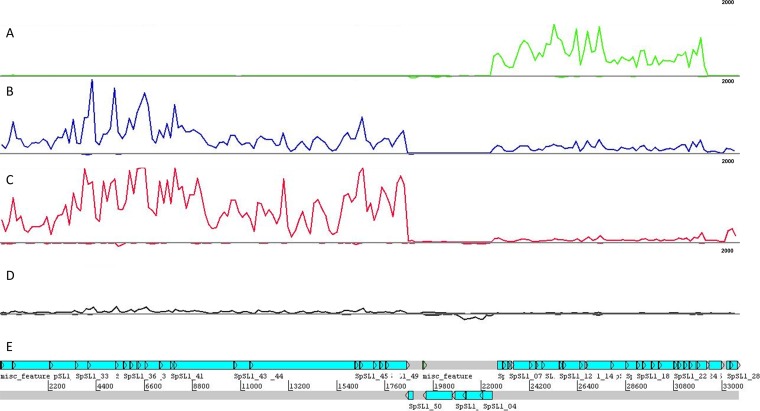
SpSL1 phage relative gene expression in lytic and lysogenic stages. (A to C) A time course of lytic infection of an *spnIII*-deleted strain at an MOI of 0.2 at 10 min (A) (green), 50 min (B) (blue), and 90 min (C) (red) after challenge. (D) Gene expression in the lysogen (black). All data are shown in terms of the normalized read coverage. (E) RNA sequencing reads were mapped to the viral conformation of the SpSL1 phage deposited in GenBank (accession number KM882824), even in the case of the lysogen (D). RNA-seq mapping and upper quartile normalization were performed using Rockhopper software. Data were visualized on BAMviewer in the Artemis tool, with the maximum number of reads being 2,000 (small label on the right of each panel). RNA-seq data were deposited at Gene Expression Omnibus GEO (accession number GSE132611).

**FIG 3:**
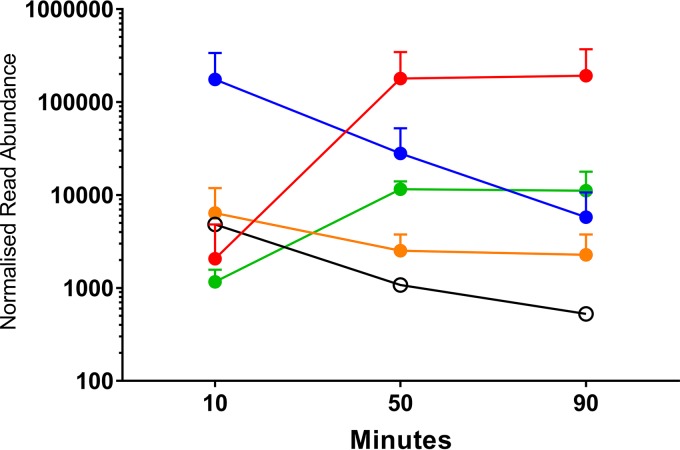
Bacteriophage SpSL1 gene expression during the lytic cycle. RNA-seq data showing the expression of phage SpSL1 at 10, 50, and 90 min postinfection of *spnIII*-negative strain FP470. The transcriptional units are numbered 1 to 5, as shown in Fig. 1A. The three early transcriptional units are operon 1 (*cds1* to *cds4*) in orange, operon 2 (*cds5* to *cds26*) in blue, and operon 5 (*cds50*) in white. The two late transcriptional units are operon 3 (*cds27* to *cds29*) in green and operon 4 (*cds30* to *cds49*) in red.

**FIG 4:**
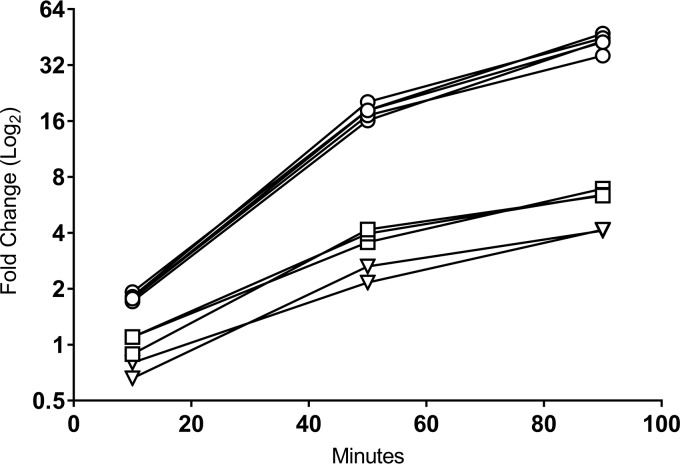
S. pneumoniae genes are upregulated in response to SpSL1 infection. RNA-seq analysis during SpSL1 infection revealed the upregulation of three S. pneumoniae transcripts preceded by an NrdR binding site and encoding the products of the anaerobic ribonucleoside triphosphate reductase operon (circles; SPD_0187 to SPD_0191, a five-gene operon), the ribonucleoside diphosphate reductase operon (squares; SPD_1041 to SPD_1043, a three-gene operon), and a hypothetical operon encoding an unknown transcriptional regulator and a conserved hypothetical protein (triangles; SPD_1594 and SPD_1595, a two-gene operon). The RNA-seq data were normalized by upper quartile gene normalization and compared with those for a noninfected control to determine the fold change in expression.

Normalized SpSL1 gene expression profiling allowed the identification of five operons (Fig. 2 and 3; Table S4). The upper quartile of gene expression normalization was used for read normalization. Upon infection, the transcription of the early replication module, including genes for DNA methyltransferases and a recombinase (operon 2, *cds5* to *cds27*), was activated immediately but then decreased in expression after 50 min (Fig. 2 and 3). *cds50* (operon 5), encoding a hypothetical protein, was actively expressed throughout the infection, but peak expression was seen at 10 min postinfection (Fig. 2 and 3). The late replication module (operon 4, *cds30* to *cds49*), containing genes for the virion packaging and structural proteins of SpSL1, including the tail fiber protein PblB, showed high levels of expression at 50 and 90 min postinfection. Similarly, expression of the operon (operon 3) encoding a hypothetical protein (*cds28*) and an endonuclease (*cds29*) increased at the 50- and 90-min time points, and these proteins are predicted to be the final proteins to be transcribed (Fig. 2 and 3). The expression profile of the lysogenic phage was also evaluated. In this case, operon 1, containing the lytic cycle repressor *cI* and the integrase gene, as well as other genes involved in lysogeny maintenance (*cds1* to *cds4*), showed the highest expression. All other phage genes were found to be actively expressed, albeit at a low level; however, it is important to note that there was the presence of antisense RNA transcription predicted throughout the SpSL1 genome (Fig. 2 and 3; Fig. S5; Table S4). The high level of expression of the *cl* repressor allowed us to identify a 5′ untranslated region of 140 bp. In order to evaluate the transcriptional response of the bacterial host cell to SpSL1 phage lytic infection, transcriptome sequencing (RNA-seq) data were also mapped onto the pneumococcal genome (Fig. 4; Table S3). Over the 90-min time course, 164 genes of the *spnDP1004III*-deleted mutant showed levels of expression significantly altered with respect to those of noninfected cells. The operons responsible for ribonucleoside triphosphate biosynthesis (SPD_0187-0191, SPD_1041-1043, and SPD_1594) were already found to be upregulated at 10 min postinfection and were by far the most highly upregulated host genes at 50 and 90 min. Of relevance, among the genes downregulated during lytic infection, we found *lytB*, encoding the pneumococcal virulence factor LytB (SPD_0853), and *sodA*, encoding manganese-dependent superoxide dismutase (SPD_0667) (Table S3). In the strain with the integrated prophage, the main downregulated gene clusters were those encoding the biosynthesis pathways of thiamine (SPD_0622-4) and pyridine (SPD_0851-3) as well as an anion ABC importer (SPD_2024-7).

### Phage restriction by the phase-variable R-M systems in S. pneumoniae.

**FIG 5:**
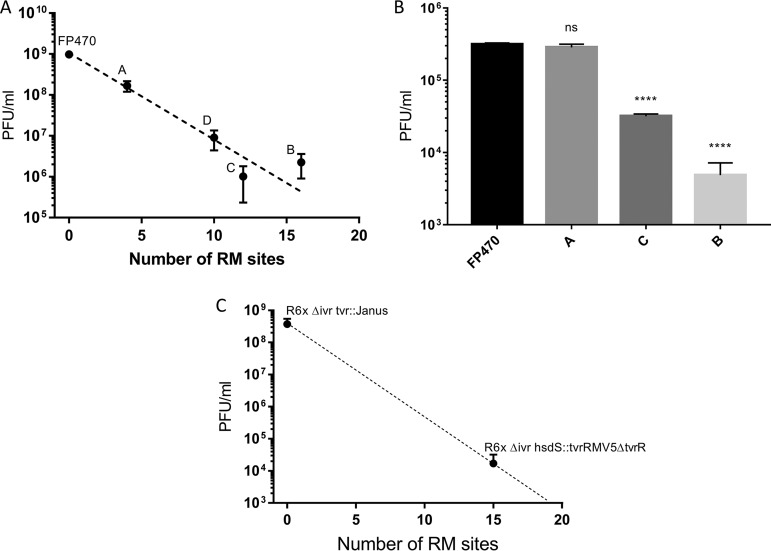
Restriction of SpSL1 by phase-variable SpnIII and SpnIV R-M systems. (A) Plaque assays results obtained using SpnDP1004III-unmethylated SpSL1 phage to infect *spnDP1004A*- to *spnDP1004D*-locked strains that express single locked copies of one of the *hsdS* alleles, with FP470 used as a control. (B) The differences observed between the control strain deleted for *spnDP1004III* (zero sites recognized) and the other mutants were statistically significant for SpSL1. ****, *P* < 0.001 by a one-way analysis of variance (ANOVA) multiple-comparison test; ns, not significant. (C) The restriction of infection of the *spnDP1004III*-deleted strain and the *spnDP1004IIIA*-locked mutant with SpnDP1004IIIA-methylated SpSL1 was not statistically significant (one-way ANOVA multiple-comparison test, *P* > 0.05), whereas the restriction of infection with the *spnDP1004IIIC* and *spnDP1004IIIB* mutants was (one-way ANOVA multiple-comparison test, *P* < 0.001) (B). Plaque assay results using SpSL1 to test the phase-variable SpnIV system showed differences between the SpnIV-knockout strain and the SpnIV R6x Δ*ivr hsdS*::*tvr*_RMV5_ Δ*tvR* recombinant (37) (Student's *t* test, *P* < 0.001).

**FIG 6:**
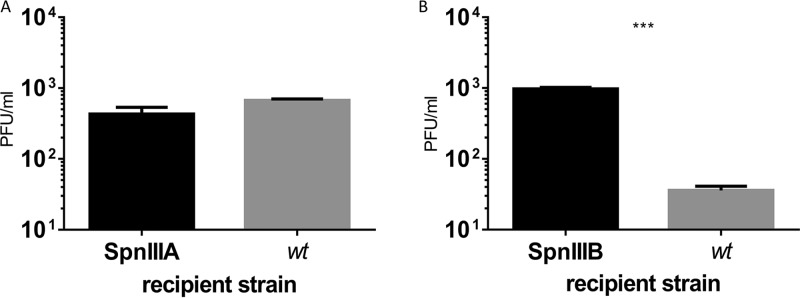
Phase-variable restriction of SpSL1 by SpnIII in a wild-type population. Plaque assays of a wt strain and *spnDP1004III*-locked mutants. The wt DP1004 strain, harboring 85% and 12% *spnDP1004IIIA*- and s*pnDP1004IIIB*-positive cells, respectively, was tested for infection by SpnIIIA-methylated phage (A) and SpnIIIB-methylated phage (B) and compared to infection of the *spnDP1004IIIA*-locked and *spnDP1004IIIB*-locked strains. (A) Equally efficient SpnIIIA-methylated phage infection of a wt host with a predominance of SpnIIIA cells and of an *spnDP1004III*-locked mutant. (B) Plaque generation of SpnIIIB-methylated phage. Unrestricted plaque formation in s*pnDP1004IIIB* cells yielded SpnIIIB-methylated phage (data of phage methylation status not shown), while infection of the wt containing 85% *spnDP1004IIIA* cells (gray bar in panel B) yielded fewer plaques (two-tailed *t* test, *P* < 0.001), and all phages obtained were SpnIIIA methylated (data not shown).

SpSL1 was assayed for its interaction with the phase-variable SpnIII and SpnIV R-M systems and its role in bacteriophage restriction. The number of target sites within the bacteriophage genome is dependent upon the variant of the SpnIII or SpnIV system present in the host cell that the bacteriophage is infecting, with a range of 4 to 16 sites being found in the different SpnIII alleles and 15 and 21 being found within the SpnIV alleles tested here. Infecting alternatively locked unencapsulated clones clearly showed different levels of restriction of SpSL1 (Fig. 5). The divergence of plaque numbers of *spnDP1004IIIA*- to *spnDP1004IIID*-locked mutant strains with respect to the *spnDP1004III*-deleted mutant was statistically significant (*P* < 0.001). The efficiency of restriction was approximately proportional to the number of methylation sites for each phase-variable variant present in the SpSL1 genome (Fig. 5A). SpnDP1004III-unmethylated phages that were successful in infecting the A- to D-locked strains were collected and assayed for the presence of methylation in the SpnIIIA to SpnIIID recognition sites through a subsequent d-LA assay. As shown with SpnIIIA-methylated SpSL1, effective methylation of the phage was demonstrated by abolishing restriction when reinfecting the *spnDP1004IIIA* strain, and restriction still occurred when infecting the *spnDP1004IIIB* and *spnDP1004IIIC* strains (Fig. 5B and C). To evaluate the impact of a functional SpnIII R-M system in a bacteriophage isolation screening protocol, a DP1004 strain with a known *spnDP1004III* allele composition (85% A, 12% B, 1.6% C, 0.75% D, 0.65% E) was challenged with an SpSL1 phage methylated at the A or B site (Fig. 6A and B). The number of plaques was greatly reduced (about 40-fold reduction, *P* < 0.001) when using an SpnIIIB-methylated phage (Fig. 6B), while no reduction was observed with SpnIIIA-methylated SpSL1 (Fig. 6A). Similarly, we tested mutants in the second pneumococcal phase-variable type I R-M system, SpnIV (37). The SpnIV-knockout strain, the R6x Δ*ivr tvr*::Janus strain, which lacked both the SpnIII and SpnIV R-M systems (Table S1), was used as a control for the phase variant R6x Δ*ivr hsdS*::*tvr*_RMV5_ Δ*tvrR* (Table S1), which expresses a functional SpnIV R-M system transferred from strain RMV5 (*tvr*_RMV5_) (37). The restriction activity of this strain showed a 10,000-fold reduction in phage activity compared with that of the control (*P* < 0.001) (Fig. 5C).

### Abortive infection mechanism.

**FIG 7:**
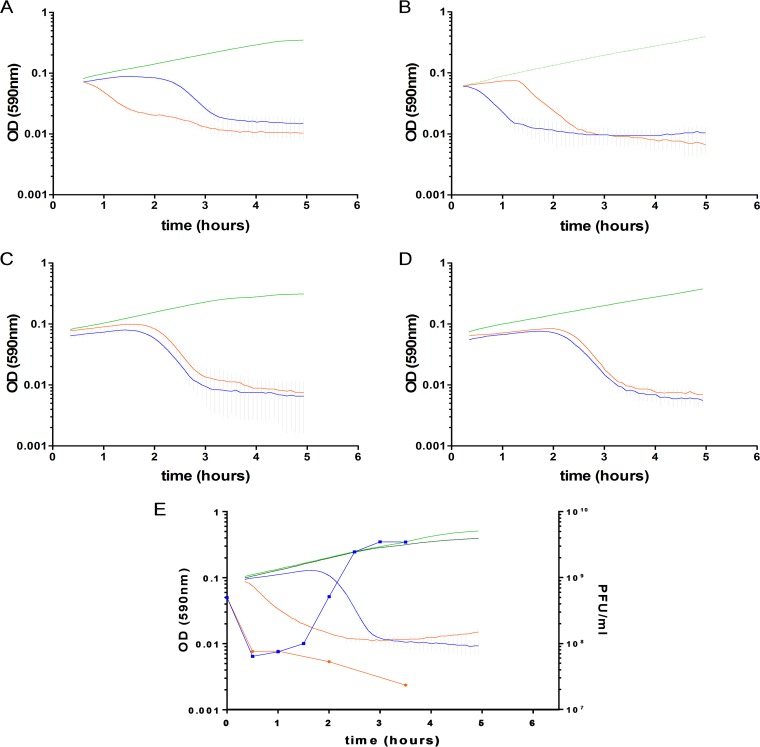
Abortive infection by the SpnIII system. Bacterial cell fate and viability were determined by the *hsdS* allele conformation and by the infecting phage genome methylation status. (A) The *spnDP1004IIIA*-locked mutant was infected with SpnIIIA-methylated (blue; MOI = 2.5) and SpnIIIA-nonmethylated (orange; MOI = 2.5) SpSL1 phages. (B) The *spnDP1004IIIB*-locked mutant was infected with SpnIIIB-methylated (orange; MOI = 2.5) and SpnIIIB-nonmethylated (blue; MOI = 2.5) SpSL1 phages. In both cases, the nonrestricted phage killed the cells after completion of the lytic cycle, while the supposedly restricted phage induced a rapid and progressive lysis. When infecting an *spnDP1004III* deletion mutant (C), the phage underwent a lytic cycle irrespective of its methylation status (SpnIIIA methylated, blue; SpnIIIB methylated, orange; MOI = 2.5). The same outcome was achieved by inactivating the restriction subunit of *spnDP1004III* alone (D). In panels A to E, uninfected bacterial strains are depicted in green. The one-step growth curves (infecting free viral particles were measured each 30 min after infection) in panel E confirmed the production of phage progeny when SpSL1 was not restricted (as with the SpnIIIA-methylated phage infecting the *spnDP1004IIIA*-locked mutant [blue lines]; light green, uninfected control) or the absence of phage replication when SpSL1 was restricted by SpnDP1004III (as with the SpnIIIA-methylated phage infecting the *spnDP1004IIIB*-locked mutant [orange lines]; dark green, uninfected control). The SpSL1 burst size was 20 PFU.

**FIG 8:**
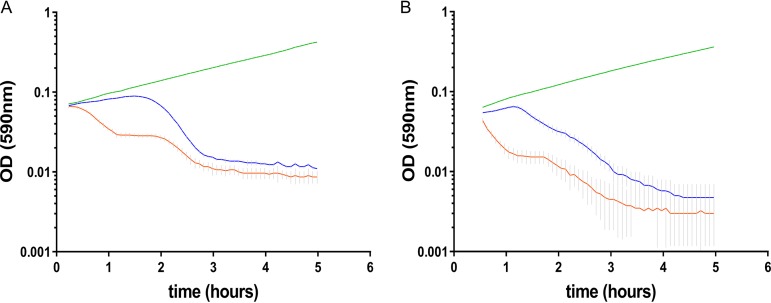
McrBC and LytA are not responsible for abortive infection. The SpnMcrBC type IV R-M system and the autolysin LytA have no effect on the Abi phenotype. Mutants for *mcrBC* (A) and *lytA* (B) were constructed in an *spnDP1004IIIA*-locked background, and the pairs of recombinant strains were infected with SpnIIIA-methylated (blue; MOI = 2.5) and SpnIIIA-unmethylated (orange; MOI = 2.5) SpSL1 phage. Uninfected controls are shown in green. Both *mcrBC* and *lytA* mutants showed nearly immediate lysis upon SpnIIIA-unmethylated phage infection, indicative of an unmodified Abi phenotype.

A mid-exponential-phase wt strain was infected with an SpnIIIA-methylated phage (multiplicity of infection [MOI] = 0.25), and the variation of the *hsdS* allele conformation in the bacterial population was evaluated at each hour for the next 4 h (Fig. S3). Despite a reduction in viable cells, no significant change in SpnIII allele frequency was identified (Fig. S3). In a similar manner, SpnIII-locked mutants were infected with SpnIIIA-methylated and SpnIIIB-methylated phages (Fig. 7A and B). CFU counts, after 55 min postinfection, showed a similar reduction of viable cells irrespective of the infecting phage’s methylation pattern (Fig. 7A and C). The involvement of the SpnIII system in the Abi phenotype was evident, in that phage recognized as self (i.e., SpnIIIA-methylated phage in an SpnIIIA-locked mutant; Fig. 7) produced lysis of the host just before 2 h (coinciding with virion release), whereas phage recognized as nonself (i.e., phage with a different methylation pattern that should be restricted) induced a progressive cell death which started immediately after the first minutes of infection (Fig. 7). The *spnDP1004III* deletion mutant lacked the Abi phenotype, showing that SpnIII itself was responsible for the cell death (Fig. 7). Mutants deleted for just the SpnIII restriction subunit (*spnDP1004* Δ*hsdR*) were also unable to undergo abortive infection. Inhibitory concentrations of chloramphenicol were found to block the abortive infection, indicative of the need for the *de novo* biosynthesis of proteins (Fig. S4). Mutants of LytA and McrBC still underwent Abi, providing further evidence that SpnIII is specifically responsible for Abi in S. pneumoniae (Fig. 8).

## DISCUSSION

To improve the methodology for the isolation of pneumococcal bacteriophages (38–40), we utilized a knockout mutant of the phase-variable type I R-M system, SpnD39III, recently described by us and others (20). As D39 and its derivatives are naturally devoid of the other phase-variable type I R-M systems (SpnIV, encoded by the *tvr* locus) (37), our new nonencapsulated recipient strain did not contain any of the phase-variable R-M systems; this likely contributed to our success in isolating the temperate bacteriophage SpSL1 from a panel of oral swab samples. This siphovirus was then investigated and used to study the underlying bacteriophage-host interactions involving the phase-variable R-M system and abortive infection.

Whole-genome analysis showed a functional cluster organization of the SpSL1 genome (Fig. 1A) similar to that of the genomes of other streptococcal prophages (6, 41–45). An interesting exception was the *cro* transcriptional regulator, which was found within the replication cluster, which is far from the lysogeny module. This finding reinforces previous evidence supporting the hypothesis that phage evolutionary exchange can take place at the level of a single gene (44, 46, 47) and contrasts with the theory of modular phage evolution (48). The absence of conserved genes of unknown function, *cg1* and *cg2*, is also noteworthy, as these genes have been described to be present in all previously characterized temperate pneumophages (6, 44). Based upon the distribution of genes, the integrase sequence homology, and the *attP* recognition sequence, SpSL1 could be included in either phage group 1, according to the classification of Romero and colleagues (44), or cluster B2, as described by Brueggemann and colleagues (6). Many regions of the genome, including the tail fiber gene, the lytic cluster, and part of the lysogeny and replication modules, showed rearrangements compared with their arrangement in other streptococcal bacteriophages. Due to the differences found between SpSL1 and other pneumococcal prophages, the primers described by Romero and colleagues (49) for the identification of temperate S. pneumoniae phages and used in a recent study (50) would not have been able to detect SpSL1.

The attachment site sequence is identical to that designated *att*_OXC_ by Romero and colleagues (44). It is present in multiple sites in the S. pneumoniae genome (see Fig. S2A, found with all supplemental material described in this article at https://doi.org/10.25392/leicester.data.8320871) and was found to be a conserved sequence belonging to four out of the five *cia*-dependent small RNAs (csRNAs) (51). These noncoding sRNAs are highly similar to each other, showing a predicted secondary structure with inverted repeats at both ends. csRNAs are present in many streptococcal genomes, suggesting a fundamental role in this group of organisms, and this likely explains the reason for them being selected as a target for phage integration (52). The expression of the csRNAs is regulated by the two-component regulatory system CiaRH (51). In S. pneumoniae, csRNAs have been shown to modulate stationary-phase autolysis (51), to affect virulence during lung infection (53), to be involved in β-lactam resistance (54), and to negatively regulate natural competence development (54, 55). However, despite the identification of some targets of the csRNAs, the molecular mechanism(s) underlying the phenotypes observed is still unknown (56). Interestingly, when analyzing the SpSL1 *attP* downstream sequence, it was observed to exhibit high nucleotide identity with csRNA2 and its right flanking region over a region of 236 bp (Fig. S2B). Therefore, in the case of phage integration at csRNA2, it would be predicted that csRNA2 would remain unaltered. Only two nucleotide changes were found between the csRNA2 and SpSL1 sequences (Fig. S2B); these were located on the loop of the predicted terminator and at the end of the small RNA sequence, suggesting an absence of significant secondary structure alterations after phage integration (Fig. S2B). Type 1 temperate phages identified previously in the genome of sequenced S. pneumoniae isolates have always been found to be integrated at the csRNA3 site alone (41, 44). The first 30 nucleotides after *att*_OXC_ in type 1 pneumophages are well conserved, with phage ϕSpn_H_1 being a good example (Fig. S2B). After phage integration, the final portion of csRNA3, corresponding to the terminator, is replaced with the end of csRNA2, producing a new chimeric sequence that maintains the secondary structure characteristic of csRNAs and that is therefore likely to be functional. Here, we report the first evidence of integration of a temperate phage at the csRNA2 site; however, this was never exclusive (Fig. S2C and D), and integration in csRNA2 was always found to be associated with another phage also integrated at csRNA3, while the reverse state was not obligatory. These observations, together with the observation of intact csRNA sites, suggested an active process of phage excision and integration occurring within the same cell. The instability of prophages in the *spnDP1004III*-deleted mutant under the growth conditions used was confirmed by the presence of free phage particles in the culture medium and also by gene expression profiling of the lysogenic strain, where genes encoding both lysis- and lysogeny-related proteins were found to be highly expressed (Fig. 2).

Lytic phage infection showed a significant impact on the global host transcriptome, with the majority of changes occurring transiently in the early stage of infection. The observed variations were typical of a metabolic stress-related response. Unlike the previous proposal for the PRD1 phage infecting E. coli (57), the amino acid uptake pathways upregulated early in E. coli were not significantly upregulated in S. pneumoniae; however, the nucleoside synthesis operons were (Fig. 4A; Table S3), potentially allowing for the greater availability of nucleosides for bacteriophage genome replication. Conversely, the transcriptional downregulation of a few other genes (namely, the manganese export, ABC transporter, and peptidoglycan biosynthesis operon genes and the gene for LytB) (Table S3) could represent the inhibition of unnecessary energy-wasting synthetic pathways in order to concentrate the host biosynthetic machinery exclusively on phage replication. The reduced expression of ABC transporters and peptidoglycan biosynthesis operons was also observed in Lactococcus lactis and Pseudomonas aeruginosa after infection with the c2 and PRR1 phages, respectively (58, 59). It is noteworthy that the previous temporal analyses of bacterial gene expression after phage infection showed the majority of changes to occur at the late stage of phage replication (57, 58, 60–62). The lysogenic phage was found to be unstable under the conditions assayed and showed expression of genes associated with both lytic and lysogenic cycles, which most likely reflects expression data from a mixed population (Fig. 2 and 3). It is therefore difficult to distinguish between the effects on host transcription of the lytic phage and the lysogenic phage alone. Of relevance is the downregulation of the pyridine biosynthesis operon (Table S3), the expression of which was also found to be reduced in L. lactis during mid to late infection of the Tuc2009 phage (60). The gene expression of SpSL1 during lytic infection shows a clear operon structure with early and late operons, as shown in most other lysogenic bacteriophages (Fig. 2 and 3). Interestingly, the *cds27* to *cds29* genes (operon 3), predicted to be involved with cell lysis, showed transcriptional regulation independent of that of the other traditional late genes, suggesting an alternative or additional regulation (Fig. 2 and 3). *cds50* (operon 5), encoding a hypothetical protein, showed independent transcriptional regulation (Fig. 2 and 3) and was less expressed than the hydrolase genes downstream or the integrase gene upstream of the lysogen.

Restriction of SpSL1 infection, as previously show with other type I R-M systems (63), was confirmed in the four mutants *spnDP1004IIIA* to *spnDP1004IIID* (Fig. 5A and B) and by testing of the SpnIV system (Fig. 5C). The efficiency of plating was reduced according to the number of sites recognized by each *hsdS* conformation in the phage genome (Fig. 5A and B), as was previously reported for other R-M systems (16, 37). Phages harvested from the *spnDP1004IIIA* mutant were found to be methylated and therefore protected in subsequent infections of the same host strain, yet they were still restricted when infecting other locked *spnIII* strains with different *hsdS* target sites (Fig. 5B). As previously hypothesized, the ability of the SpnIII system to switch between 6 active *hsdS* subunits allows the bacteria to increase their defensive repertoire by recognizing several sequence specificities without acquiring new R-M systems (Fig. 1C) (64). In addition, by use of a wild-type (wt) S. pneumoniae strain expressing multiple forms of the SpnIII system, it was shown that this could lead to the underestimation of the actual phage titer in a d-LA assay. Indeed, clear plaques could be detected only when the phage methylation matched the R-M system of the prevalent subpopulation of a heterogeneous wt strain. In contrast, when the phage methylation matched a less prevalent R-M system in the wt acceptor strain, fewer plaques than expected were detected (Fig. 6B). In our example, infection of a wt strain (85% SpnIIIA, 12% SpnIIIB) with an SpnIIIB-methylated phage yielded only plaques with SpnIIIA-methylated phage, indicating that all these new phages were produced by breaking resistance in *spnDP1004IIIA* cells. The lack of SpnIIIB-methylated phage plaques is hypothesized to derive from the fact that even if a rare SpnIIIB cell is infected, the phage progeny cannot spread to the surrounding majority of SpnIIIA cells in the soft agar (Fig. 1C). This blocks the generation of a clear plaque even in the presence of an initial infection (Fig. 6B). These observations are of relevance, considering those previous pneumophage isolations where *spnIII* wt pneumococcal strains were used (38–40). Our data showed that the successful isolation of a phage using a wt SpnIII bacterial population is influenced by (i) the free phage titer in the samples, (ii) the methylation state of phage DNA in SpnIII sites, and (iii) the SpnIII allele composition within the acceptor strain. Of course, the last two points could be bypassed using an *spnIII* deletion mutant, as shown here.

The type IV McrBC R-M system, which recognizes and cleaves between two R^m^C patterns, was previously found to be unable to efficiently restrict a methylated phage genome (65, 66); this was demonstrated by McrBC digestion of chromosomal DNA following infection by a lambda phage carrying a cloned methylase (65). Our data show that abortive infection (Abi) occurs in those bacteria that are expected to be able to restrict phage; however, our data also demonstrate that this was unaffected by the removal of the McrBC system (Fig. 8). In contrast, the SpnIII-knockout strain, as well as single mutants of the *hsdR* gene, is unable to demonstrate the Abi self-killing phenotype. Unlike previous reports (65), our research shows that it is the SpnIII system, rather than the McrBC system, that is the main determinant of Abi in S. pneumoniae. We also show the dependency of the SpnIII *hsdR* restriction enzyme for Abi in our system. Reducing the bacterial growth rate by using the bacteriostatic antibiotic chloramphenicol reduced Abi, showing that the rate of Abi is also influenced by bacterial replication (Fig. S4). Although the phase-variable restriction of foreign DNA introduced by transformation has previously been shown for both the SpnIII (20) and the SpnIV (37) systems, this is, to our knowledge, the first phase-variable type I R-M system that can induce Abi and restrict invading bacteriophages in a phase-dependent manner, thereby making the SpnIII system the key population-based armor for S. pneumoniae in the coevolution war against their natural predators.

## MATERIALS AND METHODS

### Bacterial strains and growth conditions.

Avery’s type 2 S. pneumoniae strain D39 (67, 68), its unencapsulated Rx1 derivative DP1004 (68, 69), and the mutants derived from these strains (see Table S1, found with all supplemental material described in this article at https://doi.org/10.25392/leicester.data.8320871) were routinely cultured, where not otherwise specified, in tryptic soy broth (TSB; Becton, Dickinson) at 37°C or on tryptic soy agar (TSA) plates with 3% defibrinated horse blood at 37°C in a 5% CO_2_ incubator (70–72). When performing bacteriophage sampling and propagation or when producing the bacterial lawn for the double-layer agar (d-LA) assay (73), strains were grown instead on CAT-galactose medium (Bacto Casitone, 10 g/liter; Bacto tryptone, 10 g/liter; yeast extract, 0.5 g/liter; NaCl, 5 g/liter; K_2_HPO_4_, 15 mM; 0.2% d-galactose) at 32°C up to an optical density at 590 nm (OD_590_) of 0.1 (69). CAT-galactose agar medium was supplemented with 250 U/ml of catalase (Sigma, Germany), and the plates were incubated at 37°C in 5% CO_2_.

### Mutant construction.

In both the D39 and DP1004 backgrounds, a mutant carrying a deleted *spnIII* locus (the *spnIII*-deleted mutant) and six mutants expressing only one of the possible *hsdS* variants (*spnD39IIIA* to *spnD39IIIF*, *spnDP1004IIIA* to *spnDP1004F*) (Table S1) were constructed by the gene splicing by overhang extension technique as previously described (20, 74, 75). In brief, a PCR-generated fragment that included an antibiotic selection marker (spectinomycin or kanamycin) and two flanking regions with homology to the surrounding sequence of the genomic locus to be mutated was transformed into naturally competent pneumococcal cells (75, 76). The synthetic sequences were designed to delete the two nonfunctional *hsdS* genes (SPD_0450 and SPD_0451) and the *creX* recombinase (SPD_0452) (77) to prevent any further rearrangement leading to changes in the six variants of the enzyme. The primers used to generate such mutants of the DP1004 strain were the same as those used for D39 and are published elsewhere (20). The primers used to build the PCR products for the deletion of the whole *spnIII* (from SPD_0449 to SPD_0455) and SpnMcrBC (SPD_1108-9) systems are listed in Table S1. All mutants were confirmed by Sanger sequencing (Eurofins Genomics, Germany). SpnIV mutants were constructed by Kwun et al. (37).

### Sample collection.

Oral swab samples were collected from healthy adult volunteers at the University of Leicester, resuspended in 5 ml of SM buffer (10 mM MgSO_4_, 100 mM NaCl, 50 mM Tris-HCl, pH 7.5), and stored at 4°C with protection from light (78, 79). A portion of each of the samples was stored at −80°C with 10% glycerol. Sample collection and storage conditions were approved by the Departmental Research Ethics Office of the University of Leicester (authorization mro5-5d40, 21 July 2014). All the experiments were done in accordance with national and institutional guidelines.

### S. pneumoniae bacteriophage isolation method.

The two *spnIII*-deleted mutants FP486 and FP470 were used as hosts for propagation. The oral swab samples were added at 1:100 to mid-exponential-phase growing cultures. Overnight enrichments were centrifuged, filtered (0.22-μm-pore-size membrane), and inoculated into a fresh bacterial culture for three consecutive days. On each day, the supernatants were spot assayed on a CAT-galactose soft medium lawn plate to check for plaques, confirming the presence of bacteriophages. The double-layer agar (d-LA) assay was then performed using any positive samples. Collection of a single plaque into SM buffer and propagation were repeated several times in order to isolate a single clone of the phage. In order to identify the phage’s natural host, the oral swab sample was plated on a TSA-blood plate, and single colonies were isolated and propagated. Subsequently, each strain underwent PCR screening for the presence of the phage using primers LF_83 and LF_84 (Table S2).

### Lysogen isolation.

Lysogens of FP470 were generated by plating 1 × 10^4^ CFU on a CAT-galactose double-layer agar plate, with the overlay containing 10^8^ PFU/ml of the bacteriophage. The bacterial clones grown on the plate were propagated for several rounds on TSA. The presence of lysogenic phage and analyses of insertion sites were evaluated by PCR using the primers listed in Table S1.

### Electron microscopy.

A sterile high-titer phage sample (1 × 10^9^ PFU/ml) was purified by serial centrifugation at 20,000 × *g* for 60 min and resuspended in ammonium acetate solution. The suspension was then adsorbed onto a hydrophilic (freshly glow discharged) carbon-coated Pioloform film-coated copper grid (Agar Scientific) and negatively stained with 1% uranyl acetate. Sample visualization was performed on a JEOL 1400 transmission electron microscope (TEM) with an accelerating voltage of 80 kV, and images were captured using a Mageview III digital camera with iTEM software (Olympus).

### Identification of phage structural proteins.

Phage was purified as described above for the electron microscopy methodology. In-gel trypsin digestion of the purified phage SpSL1 followed by liquid chromatography coupled with tandem mass spectrometry (LC-MS/MS; LTQ-Orbitrap-Velos-ETD mass spectrometer) was performed at the local proteomics facility (PNACL, University of Leicester, Leicester, UK). The resulting peptide sequences were searched (MS/MS ion search; Mascot, version 2.2.04, algorithm; Matrix Science) against the sequences in the UniProtKB–Swiss-Prot and NCBI protein databases.

### Phage methodologies.

Adsorption was measured by PFU titration from the supernatant at various time points following inoculation of the phage into an FP470 growing culture (OD_590_ = 0.1, MOI = 0.1). To evaluate the restriction and methylation activities of the SpnDP1004III system, d-LA assays were performed using SpnDP1004III-unmethylated phage, derived from an *spnDP1004III*-deleted mutant, at several dilutions to infect all six strains, *spnDP1004IIIA* to *spnDP1004IIIF*. The same experiment was also performed in the presence of 125 ng/ml of competence-stimulating peptide (CSP). Plaques were counted and collected in SM buffer, and phage was propagated in the same mutant used for the d-LA assay in order to obtain the 10^9^-PFU/ml titers needed for further experiments. An SpnIIIA-methylated SpSL1 phage was then used to infect each of the *spnDP1004IIIA* to *spnDP1004IIIF* mutants and the *spnDP1004III*-deleted strain as a control. Prior to phage infection, the bacterial culture was sampled and analyzed with an allele scan protocol (20).

### Sequencing and bioinformatics analysis.

Bacteriophages were concentrated by centrifugation for 3 h at 4°C in a TH-641 rotor (Thermo Scientific) at 164,000 × *g*. Phage DNA purification was performed using the phenol-chloroform extraction technique. Sequencing of the phage genome was performed by GenProbio (Parma, Italy) using a personal genome machine sequencer (IonTorrent) and by the Norwegian Sequencing Center (Oslo, Norway) with a MiSeq platform (Illumina). Reads were assembled using both the MIRA (version 3.9.18) and Velvet (version 1.2.10) programs, and the results were combined. Genome edges encompassing the phage *cos* site were confirmed by Sanger sequencing using primers LF83 and LF84 (Table S1). The complete sequence was annotated with RAST, manually refined using the BLASTp (NCBI) and Pfam (EMBL-EBI) programs, and deposited in GenBank with accession number KM882824.

### Methylome analysis.

To evaluate the activity of the m5C methyltransferase (MTase) carried by SpSL1, DNA was treated with sodium bisulfite, which converts unmethylated cytosine (C) to thymine (T), before Illumina sequencing by the Norwegian Sequencing Center (Oslo, Norway). The reads obtained were aligned against two versions of the phage sequence in which C’s were replaced with T’s and guanines (G’s) were replaced with adenines (A’s), respectively. Polymorphic changes between T’s and C’s or A’s and G’s with a frequency over 50% were then retrieved using the Mosaik Aligner suite (The MarthLab, USA) and scored as methylated. Comparison of sequences adjacent to the methylated bases allowed us to obtain the m5C MTase recognition pattern.

### Gene expression analysis.

For gene expression analysis, pneumococcal strains were grown in CAT-galactose to mid-log phase (OD_590_, approximately 0.15) and infected with the SpSL1 phage (MOI = 0.2). After the 10-, 50-, and 90-min time points, 10 ml of cells was added to 2 ml of an ice-cold 95% ethanol–5% phenol solution, before centrifugation at 4,000 rpm for 10 min. The supernatant was removed, and the pellets were stored at −80°C until processing. Excluding the infection and time course sampling, the same procedure was followed for the strain with an integrated SpSL1. A noninfected sample was included in the analysis, and three independent replicas were collected for each condition. For RNA extraction, the pellets were resuspended in 50 μl TE (Tris-EDTA; pH 8) with 3 mg/ml lysozyme and incubated at 37°C for 20 min to lyse the cells. A Maxwell 16 LEV simplyRNA cells kit (Promega) was then used along with a Maxwell 16 LEV instrument (Promega) for RNA extraction. RNA samples were processed with a ScriptSeq complete kit for bacteria (CamBio), which includes an rRNA depletion step, and sequenced with a MiSeq system (Illumina) at the University of Leicester (Leicester, UK). RNA-seq fastq files were gently trimmed using the Trimmomatic (version 0.30) tool. Read mapping to the S. pneumoniae D39 genome (GenBank accession number NC_008533) and to SpSL1 phage (GenBank accession number KM882824), transcript abundance quantification and the upper quartile of gene expression normalization, and differential expression analysis were carried out using Rockhopper software (version 2.0.2). Differential analysis of the S. pneumoniae D39 genome was carried out against a noninfected control. Differentially expressed genes were further filtered by discarding those with a nonstatistically significant false discovery rate (*q*) value (>0.01) and values below a 2 log_2_-fold increase or decrease in transcription (75).

### Allele quantification.

Quantification of the six possible *hsdS* variants in wt strains was performed as previously described (20). In brief, a region common to all of the possible *hsdS* conformations was PCR amplified, with one of the two primers being fluorescently tagged. A double digestion of the PCR products, using restriction enzymes DraI and PleI (New England Biolabs), allowed for the generation of labeled DNA fragments of different lengths, one specifically for each of the *hsdS* variant forms. GeneScan analysis on an ABI Prism gene analyzer was then performed for relative quantification of the six allelic forms.

### Data availability.

The complete SpSL1 sequence was deposited in GenBank with accession number KM882824. RNA sequencing data were deposited at the Gene Expression Omnibus database with accession number GSE132611.
